# Complete Mitochondrial Genome of Two Ectoparasitic Capsalids (Platyhelminthes: Monogenea: Monopisthocotylea): Gene Content, Composition, and Rearrangement

**DOI:** 10.3390/genes13081376

**Published:** 2022-08-01

**Authors:** Changping Yang, Binbin Shan, Yan Liu, Liangming Wang, Qiaer Wu, Zhengli Luo, Dianrong Sun

**Affiliations:** 1 Key Laboratory of Marine Ranching, Ministry of Agriculture and Rural Affairs, South China Sea Fisheries Research Institute, Chinese Academy of Fisheries Sciences, Guangzhou 510300, China; yangchangping@scsfri.ac.cn (C.Y.); shanbinbin@scsfri.ac.cn (B.S.); liuyan@scsfri.ac.cn (Y.L.); wangliangming@scsfri.ac.cn (L.W.); wqe66@163.com (Q.W.); luohua0303@163.com (Z.L.); 2 Guangdong Provincial Key Laboratory for Healthy and Safe Aquaculture, School of Life Science, South China Normal University, Guangzhou 510631, China; 3 School of Fisheries of Zhejiang Ocean University, Zhoushan 316022, China

**Keywords:** mitochondrial genome, Capsalidae, monogenean, gene rearrangement, phylogenetic analysis

## Abstract

The capsalid monogeneans are important pathogens that generally infect marine fishes and have a substantial impact on fish welfare in aquaculture systems worldwide. However, the current mitogenome information on capsalids has received little attention, limiting the understanding of their evolution and phylogenetic relationships with other monogeneans. This paper reports the complete mitochondrial genomes of *Capsala katsuwoni* and *Capsala martinieri* for the first time, which we obtained using a next-generation sequencing method. The mitogenomes of *C. katsuwoni* and *C. martinieri* are 13,265 and 13,984 bp in length, respectively. Both species contain the typical 12 protein-coding genes, 2 ribosomal RNA genes, 22 transfer RNA genes, and a control region. The genome compositions show a moderate A+T bias (66.5% and 63.9% for *C. katsuwoni* and *C. martinieri*, respectively) and exhibit a negative AT skew but a positive GC skew in both species. One gene block rearrangement was found in *C. katsuwoni* in comparison with other capsalid species. Instead of being basal to the Gyrodactylidea and Dactylogyridea or being clustered with Dactylogyridea, all species of Capsalidea are grouped into a monophyletic clade. Our results clarify the gene rearrangement process and evolutionary status of Capsalidae and lay a foundation for further phylogenetic studies of monogeneans.

## 1. Introduction

Monogeneans are a class of important helminth parasites that are commonly found on the gills, skin, branchiostegal membranes, or buccal cavities of marine and freshwater fishes [1]. Monogeneans have direct life cycles and multiple reproductive strategies, resulting in easy propagation in natural and aquaculture environments [2]. Poor fish health, retarded fish growth, and reduced value of the market product are often caused by monogenean flukes feeding on the cells and mucus of host fish [3]. The *Capsala*, which was established by Bosc (1811), is a genus of the family Capsalidae Baird, 1853, which comprises 9 subfamilies, 48 genera, and about 200 species [4,5]. Currently, 22 species of *Capsala* have been recognized [6], among which *C. martinierei* is considered to be the largest (up to 3 cm) known monogenean species.

The evolutionary relationships in monogeneans remain debatable. For example, on the basis of comprehensive morphological characteristics, Capsalidea was considered basal to the Gyrodactylidea and Dactylogyridea [7]. However, it was inferred to be phylogenetically closely related to the Gyrodactylidea and Dactylogyridea based on the evidence of the 28S rRNA [8], 18S rRNA [9], and mitochondrial genes [10]. For the family Capsalidae, its monophyly is currently supported by the presence of accessory sclerites or modified hooklets on the haptor, providing a synapomorphy for the family [11]. Despite being studied for nearly 230 years [4], many aspects of the classification, systematics, validity, and phylogenetic position (for most species) of the capsalid species taxon are still not resolved. For example, the genera *Neobenedenia* and *Benedenia*, which belong to the subfamily Benedeniinae, were revealed to be paraphyletic categories, by analyzing the large subunit rDNA sequence data [5].

Though the characterization of *Capsala* dates back to 1811, this genus was not recognized in China until 2019 [12]. *C. martinierei* was first reported on the surface of *Mola mola* in Chili, with the following morphological characteristics: haptoral accessory sclerites absent; dorsomarginal body sclerites consist of multiple scattered bicuspid and multicuspid sclerites [4]. *C. katsuwoni* was first recorded on the gills of *Katsuwonus pelamis* and was characterized by a haptor diameter accounting for approximately 20% of the body’s length, haptoral accessory sclerites being bifid at one end, and the presence of a small finger-like fringe on the edge of the haptoral marginal valve [4,5,6]. Despite the clarity of the classification and morphology for most capsalid species, only a limited number of genomic resources exist for this family, which limits our understanding of its molecular evolution and phylogenetic relationships.

The mitochondrion is a fundamental place for respiration and energy production and thus plays an important role in cell metabolism [13]. Owing to the abundance of mitochondria in animal tissues, maternal inheritance, and the absence of introns, mitochondrial genomes have become a powerful tool in population genetics, phylogenetics, and diagnostics [14,15,16]. The mt genomes of monogeneans usually have a set of 36 genes, including 12 protein-coding genes, 22 transfer RNA (tRNA) genes, and 2 ribosomal RNA (rRNA) genes, which are organized and oriented in different ways [17]. This diversity makes them a valuable alternative tool for species identification and phylogenetic studies at the genomic level. Several mt genomes of capsalids have been reported including *Capsala pricei* [18], *Benedenia Diesing* [19], *Neobenedenia melleni* [20], and *Capsaloides cristatus* [21]. The phylogenetic position of the Capsalidae was deduced by either morphological characters or molecular markers.

In this study, the complete mitochondrial genomes of two capsalid monogeneans on fish found in the South China Sea were newly sequenced. We described the details of genome assembly, annotations, codon usage, and amino acid usage. The available complete mitogenomes of monogenean species, retrieved from the GenBank database, provided insight into the phylogenetic relationship between capsalids and other monogenean species. These results will help us better understand the gene arrangement features of monogenean mitogenomes and lay the foundation for further phylogenetic study of this highly diverse flatworm group.

## 2. Materials and Methods

### 2.1. Sampling and DNA Extraction

During two fishery surveys in the South China Sea, 10 specimens of *C. martinierei* were collected from the skin of *Mola mola* Linnaeus, 1758 on 10 February 2021 in the Zhongsha Sea area (13°52′ N, 110°98′ E), and 22 specimens of *C. katsuwoni* were collected from the gills of *Auxis thazard* (Lacepède, 1800) on 15 May 2018 in the Nansha Sea area (9°30′ N, 114°00′ E). After collection, the specimens of the two capsalid parasites were preserved in 95% ethanol at −20 °C for long-term storage. Whole-genome DNA was extracted from one specimen for each parasite species using a Steady Pure Universal Genomic DNA Extraction Kit (Accurate Biotechnology, Changsha, China) following the manufacturer’s instructions. DNA quality was evaluated through electrophoresis in a 1% agarose gel and a NanoPhotometer^®^ spectrophotometer (IMPLEN, Westlake Village, CA, USA).

### 2.2. Mitochondrial Genome Sequencing and Assembling

The library was constructed using a TruseqTM RNA sample Prep Kit (Illumina, San Diego, CA, USA) with 1 μg DNA, which was fragmented into 300–500 bp by a Covaris M220. The library was then sequenced using an Illumina Hiseq platform. High-quality clean data were obtained by filtering out low-quality reads and duplicated reads. Clean data were assembled into optimal contigs by the de novo assembler, NOVOPlasty (https://github.com/ndierckx/NOVOPlasty, accessed on 10 April 2021). The gene map was generated with the online program OGDraw v1.2 [22].

### 2.3. Sequence Annotation and Analysis

The complete mitogenome was annotated using the software Sequin (version 15.10, http://www.ncbi.nlm.nih.gov/Sequin/, accessed on 18 April 2021). The boundaries of the ribosomal RNA genes were performed using NCBI-BLAST (http://blast.ncbi.nlm.nih.gov, accessed on 20 April 2021). Codon usage, amino acid proportion, and relative synonymous codon usage for the 12 protein-encoding genes of the two studied monogenean species were calculated using PhyloSuite. Strand asymmetry was calculated using the formulae: AT-skew = (A − T)/(A + T); GC-skew = (G − C)/(G + C) [23].

### 2.4. Gene Rearrangement Analysis and Phylogenomic Reconstruction

The gene rearrangements and phylogenomic analysis were analyzed by comparison of the two newly sequenced mitochondrial genomes of *Capsala* with 24 species of monogeneans retrieved from GenBank, including 8 species of Gyrodactylidae, 4 species of Capsalidae, 2 species of Tetraonchoididae, 2 species of Diplectanidae, 2 species of Ancylodiscoididae, 4 species of Ancyrocephalidae, and 2 species of Dactylogyridea (Table 1). *Schistosoma japonicum* from Diplostomida (Digenea) was used as an outgroup species for phylogenomic analysis.

MAFFT was used to perform the sequence alignment [24], and then the data sets were trimmed by trimAl [25]. The concatenated set of nucleotide sequences was used for phylogenetic analysis, which was performed with the BI and ML methods using MrBayes v3.2.6 [26]. The optimal evolution model was GTR + I + G in the jModelTest v2.1.7 [27], and the maximum-likelihood method was used to infer the phylogenetic relationship with 1000 bootstrap replicates in MEGA 6.0 [28]. Bayesian inference (BI) was performed using Mrbayes v3.2 [29]. In the Markov chain Monte Carlo (MCMC) analysis, we ran 1 × 10^8^ generations. Samples were taken every 1000 generations, and the first 25% were discarded as burn-in. The stationarity was achieved when the average standard deviation of the splitting frequency remained below 0.01. The resulting phylogenetic trees were visualized in FigTree v1.4.2.

## 3. Results and Discussion

### 3.1. Genome Organization

**Figure 1:**
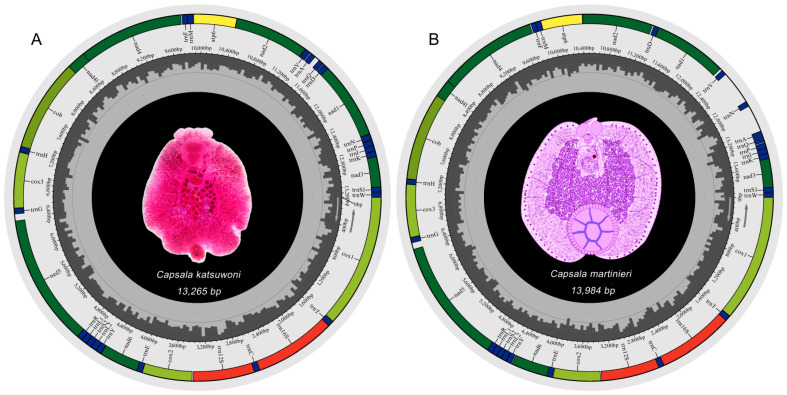
Mitochondrial genome maps of *Capsala katsuwoni* (**A**) and *Capsala martinierei* (**B**). Both photographs in the genome maps are ventral view of the whole monogenean bodies. Map A is a microscopy photo of one stained specimen of *C. katsuwoni,* and map B is a hand-painted image of *C. martinierei* colored with Photoshop. Protein-coding genes (PCGs) are color-coded (*cox*: light green, *nad*: dark green, *cob*: gray-green, *atp*: yellow), tRNA genes are in blue, and rRNA genes are in red. Abbreviations of PCGs are: *cox*1–3 for cytochrome oxidase subunits 1–3; nad1–6 and nad4L for NADH dehydrogenase subunits 1–6 and 4 L, respectively; *cob* for cytochrome *b*; atp6 for ATP synthase subunits 6; and rrn12 and rrn16 for small and large rRNA subunits, respectively.

The mitochondrial genomes of *C. katsuwoni* and *C. martinierei* are typical circular molecules that are 13,265 and 13,984 bp in length, respectively (Figure 1). They were deposited in GenBank under accession numbers OL884727 and OL790148, respectively. The length of the *C. martinierei* mitogenome is close to the boundaries of those previously reported in Capsalidae species (13,270 bp for *Neobenedenia melleni* to 13,948 bp for *Capsaloides cristatus*) [20,21]. The mitogenomes of both studied species contain 12 protein-coding genes (PCGs), 2 rRNAs, and 22 tRNAs, which is in accordance with those of other genera in Capsalidae [18]. In accordance with previously studied monogenean species [20,30], the atp8 gene is absent. There are slight differences between *C. katsuwoni* and *C. martinierei* in the sizes of PCGs (10,026 vs. 9966 bp), rRNAs (1662 vs. 1665 bp), and tRNAs (1429 vs. 1436 bp, Table 2). Their overlapping sequences and intergenic sequences are also slightly different. *C. katsuwoni* has 7 overlapping sequences ranging from 1 to 36 bp and 20 intergenic sequences ranging from 1 to 80 bp. On the other hand, *C. martinierei* has 7 overlapping sequences ranging from 1 to 35 bp and 18 intergenic sequences ranging from 1 to 413 bp. The longer genomes of *C. martinierei* should be ascribed to two long intergenic fragments between trnV-trnN and trnN-trnA. 

**Figure 2:**
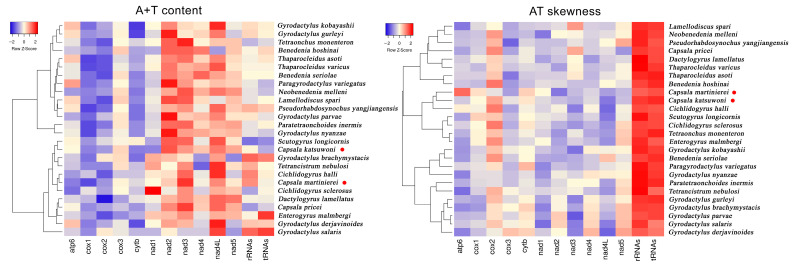
Hierarchical clustering maps of the A+T content and AT skewness of 13 mitogenomic elements among the 26 selected monogeneans. The red dots represent the two newly sequenced capsalid species.

The nucleotide distributions are also different between *C. martinierei* and *C. katsuwoni*. Namely, *C. martinierei* contains less T than *C. katsuwoni*, while the former has a higher G+C content than the latter. The GC skewness of mitochondrial genomes is slightly positive (0.13 and 0.06), while the AT skewness is negative (−0.24 and −0.19) for *C. katsuwoni* and *C. martinierei*, respectively (Table 2). Compared with previously reported capsalid monogeneans, the A+T contents of the two newly sequenced *Capsala* species are relatively low, while their AT skewness is similar to that of *Benedenia hoshinai* (Figure 2, Tables S1 and S2). In most monogenean mitogenomes, the strand skew biases are found to have a negative AT skew and positive GC skew [31]. The strand skew biases of monogenean mitogenomes varies between −0.45 and −0.01, and 0.05 and 0.50 for AT and GC, respectively [32]. For both sequenced mitogenomes of the present study, the PCGs exhibited the highest negative AT skewness in comparison with tRNAs and rRNAs, in accordance with those of other previously recorded monogenean species [33].

### 3.2. Protein Coding Genes and Codon Usage

The 12 protein-coding genes, accounting for 75.6% and 71.3% of the whole mitochondrial genome, encode 2998 and 3140 amino acids of *C. katsuwoni* and *C. martinierei*, respectively (Table 2, Tables S3 and S4). All protein-coding genes are coded on a majority strand (J-strand). The initiation codons and termination codons are different between species (Table 3). There are three initiation codons (ATG, ATA, and GTG) and three termination codons (TAA, TAG, and TGA) in the mitochondrial genome of *C. martinierei*, while only two initiation codons (ATG and GTG) and two termination codons (TAA and TAG) were found in the mitochondrial genome of *C. katsuwoni*. The most common initiation codon is ATG, which occurs in ten and nine genes in *C. martinierei* and *C. katsuwoni* mitogenomes, respectively. The starting codon is ATA in nad4 and GTG in nad3 in the mitogenome of *C. martinierei*. In the *C. katsuwoni* mitogenome, nad2, nad4, and nad4 L use GTG as an initiation codon. The most common stop codon is TAG, which occurs in seven genes in the *C. martinierei* mitogenome. Four genes (cox1, nad6, cox3, and cob) use TAA as the stop codon. The least frequent termination codon in *C. martinierei* mitogenome is TGA, which was only seen in nad1. In *C. katsuwoni* mitogenome, half of the genes use TAA as a stop codon, while the other half use TAG.

**Figure 3:**
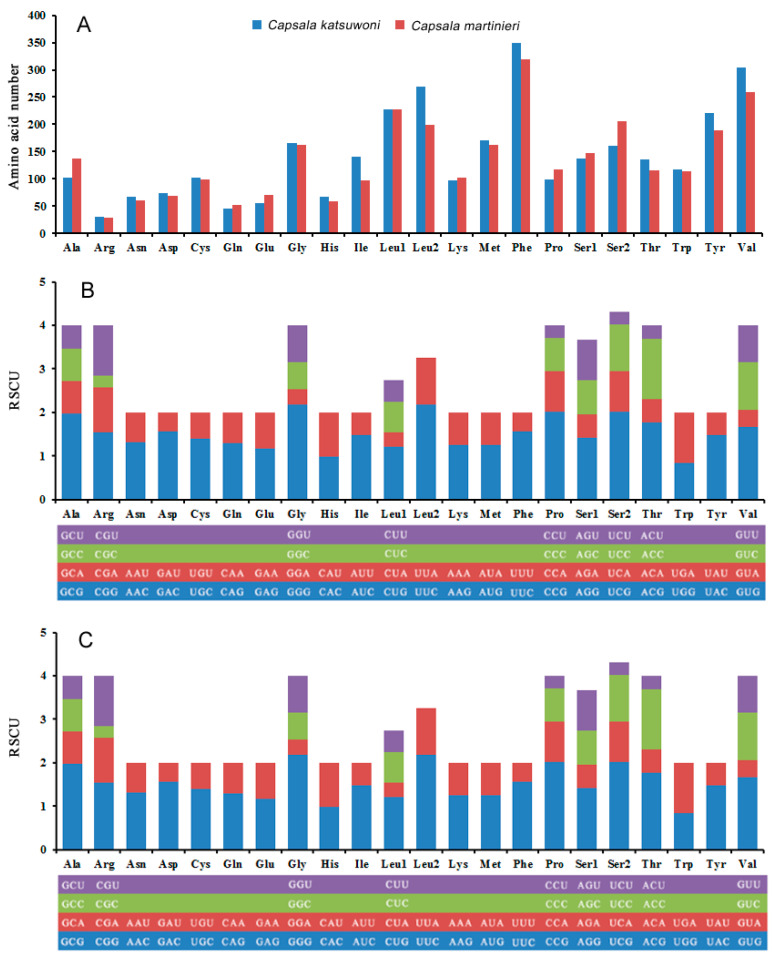
Amino acid composition in *Capsala katsuwoni* and *Capsala martinieri* mitogenome (**A**); relative synonymous codon usage in *C. katsuwoni* (**B**) and *C. martinieri* (**C**) mitogenome. The box below the bar chart represents all codons encoding each amino acid, and the height of the column above represents the sum of all RSCU values.

The most common codon used in protein-coding genes is leucine (Leu1 + Leu2), while arginine is the least used codon in both *Capsala* species (Figure 3A). This is different from the *Benedenia diesing* mitogenome, which uses glutamine the least in protein-coding genes (Baeza, Sepúlveda, et al., 2019). The relative synonymous codon usage (RSCU) values for the 12 PCGs are shown in Figure 3B,C and Tables S3 and S4. The usage of both two- and four-fold degenerate codons is biased toward codons abundant in T or A, which is in accordance with other species of Capsalidae [33].

### 3.3. Transfer RNA Genes and Ribosomal RNA

Like most monogenean mitogenomes, the *Capsala* mitogenome contains a set of 22 tRNA genes [31,32,33], and all of them are encoded on the majority strand (Table 2). The tRNA genes range in size from 42 bp (Ser1 in *C. martinierei*) to 68 bp (Cys in *C. martinierei* and Phe in *C. katsuwoni*), and the total lengths were 1429 and 1436 bp in *C. katsuwoni* and *C. martinierei*, respectively. The anticodons of all the tRNA genes in two newly sequenced species are consistent with those found in closely related monogenean mitogenomes [20], with the exception of *C. martinierei,* which exhibits the anticodon AGC instead of GCT in the trnS1 gene. The locations of the rRNAs are the same in both *Capsala* species; the large rRNA subunit is between trnT and trnC, while the small rRNA subunit is located close to the large rRNA subunit, between trnC and cox2. The locations of both rRNA subunits are conserved in the previously reported monogenean species [18,20,21], with the exception of two Ancylodiscoididae species (*Thaparocleidus asoti* and *Thaparocleidus varicus*) [31].

### 3.4. Gene Rearrangement

**Figure 4:**
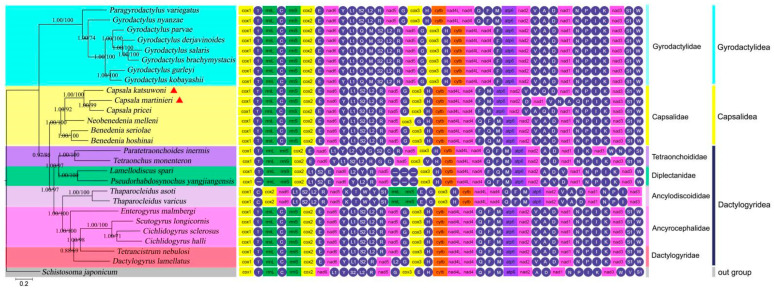
Phylogram reconstructed using 26 monogenean mitogenomes of seven families, with gene order displayed to the right of the tree. The phylogenetic tree was inferred from the nucleotide sequences of 12 mitogenome PCGs using ML and BI methods. Statistical support values of BI and ML are shown by the nodes (left/right). Scale bar corresponds to the estimated number of substitutions per site. Monogenean families and orders are shown in different colors. The newly determined species are emphasized by triangles.

For a better comparison of the gene order among monogenean species, we extracted and visualized the previously reported and sequenced mitogenomes for 24 species of Monogenea. This resulted in a set of 13 unique gene orders (Figure 4). In general, gene orders within Capsalidae mt genomes are relatively conserved. The newly sequenced *C. katsuwoni* genome has an identical gene order to another reported *Capsala* species (*C. pricei*). The other species of Capsalidae mt genomes exhibit only minor variations in the tRNA order. In both *Benedenia* species, the trnQ gene occurs between trnF and trnM, while the same gene is located between trnA and trnD in *C. katsuwoni*. The gene order of *C. katsuwoni* is remarkably similar to those of Ancyrocephalidae species, and the transformational pathway from the former to the latter requires only one transposition of trnQ. In comparison with *C. katsuwoni*, *C. martinierei* exhibited a significant rearrangement in one gene block: from trnV-trnA-trnQ-trnD-nad1-trnN to trnD-nad1-trnV-trnN-trnA-trnQ.

Conserved gene order is a typical feature of mitogenomes [34], which is the case of Garodyctylidae and Ancyrocephalidae in our analysis. Our results suggested that extensive gene order rearrangement occurred in the *Capsala* genus, which further confirmed the hypothesis that gene order in monogeneans is evolving at a relatively rapid rate [33]. Several commonly used mechanisms, including duplication-random loss, duplication-nonrandom loss, and recombination [35,36,37], have been proposed to explain the gene rearrangements of mitogenomes. Here, we only observed recombination in *C. martinierei* mitogenome compared with *C. katsuwoni*, where trnD-nad1 is translocated upstream of trnV, and trnN is translocated to the position of the trnV and trnA junction. The underlying mechanism of this recombination among *Capsala* species needs further study. As for the gene order in monogeneans, the evolution of mitogenomic gene order arrangements is generally continuous, with the exception of the *Thaparocleidus* genus. Our conclusions should be interpreted with more available monogenean mitogenomes to produce reliable evolutionary signals.

### 3.5. Phylogenetic Analyses

The phylogenetic analysis included twenty-six species from seven families of the Monogenea and one outgroup species (*Schistosoma japonicum*) (Figure 4). Overall, the monogeneans divide into two clades: one containing the orders of Capsalidea and Dactylogyridea, and the other with only Gyrodactylidea species. This classification was chosen because, from the view of morphology, both Dactylogyridea and Capsalidea have 14 marginal hooklets on the haptor, while Gyrodactylidea has 16 hooklets [38]. This analysis is in agreement with those of previous studies that used mitogenomic data to reconstruct the phylogenetic relationships among monogeneans [39].

The genetic distances between families from the Capsalidea and Dactylogyridea orders are controversial. Zhang et al. reported that two dactylogyrid monogeneans formed a sister group with the three capsalid species, even closer than those species from Diplectanidae and other families of Dactylogyridea [10]. With two newly sequenced *Capsala* species, our results clearly classify Capsalidae into Capsalidea and Diplectanidae and Dactylogyridae into Dactylogyridea. The closer phylogenetic relationships between the Dactylogyridae and Diplectanidae than either of the two with Capsalidae are supported by several morphological- and molecular-data-based studies, including spermatozoon ultrastructure, comprehensive morphological characters, 28S rRNA, and 18S rRNA [35,40,41]. Hence, our results provided comprehensive phylogenetic relationships among the monogeneans with more confidence.

Additionally, we found *C. martinierei* clusters with *C. pricei*, and further forms a sister group with *C. katsuwoni*, which is inconsistent with the gene orders of these three species. *C. katsuwoni* shows the same gene order as *C. pricei*, while the recombination of one gene block occurs in *C. martinierei*. Therefore, we realized that gene order alone cannot be used to analyze the phylogenetic relationships of monogeneans.

## 4. Conclusions

In the present study, we sequenced and analyzed the mitogenomes of two capsalid species, which are common parasites on oceanic fishes. In summary, the mitogenome of *C. katsuwoni* shows a relatively conserved gene architecture, while extensive gene order rearrangement occurred in *C. martinierei*. The monophyly of Capsalidea was strongly supported by the phylogenetic analysis based on the PCG data. Our results provided useful information for further understanding the gene rearrangement process, phylogenetics, and evolution of monogenean parasites.

## Figures and Tables

**Table 1:** List and composition of monogenean species analyzed in this study with their GenBank accession numbers.

Order	Family	Species	Full Length	A%	T%	G%	C%	GenBank No.
Capsalidea	Capsalidae	*Capsala katsuwoni*	13,265	25.4	41.1	18.9	14.6	OL884727
Capsalidea	Capsalidae	*Capsala martinierei*	13,984	25.8	38.2	19.1	17.0	OL790148
Capsalidea	Capsalidae	*Capsala pricei*	13,851	26.1	43.2	17.3	13.4	MN746360
Capsalidea	Capsalidae	*Benedenia hoshinai*	13,554	28.7	45.4	14.9	11.0	NC_014591
Capsalidea	Capsalidae	*Benedenia seriolae*	13,498	28.9	46.7	14.6	9.8	AP019637
Capsalidea	Capsalidae	*Neobenedenia melleni*	13,270	30.7	45.2	14.7	9.4	JQ038228
Dactylogyridea	Ancyrocephalidae	*Cichlidogyrus halli*	15,047	25.4	38.0	13.2	23.2	MG970255
Dactylogyridea	Ancyrocephalidae	*Cichlidogyrus sclerosus*	15,052	24.9	40.5	11.7	22.9	JQ038226
Dactylogyridea	Dactylogyridae	*Dactylogyrus lamellatus*	15,187	27.5	43.1	9.9	19.5	KR871673
Dactylogyridea	Ancyrocephalidae	*Enterogyrus malmbergi*	14,107	27.0	42.0	10.2	20.8	NC_048529
Gyrodactylidea	Gyrodactylidae	*Gyrodactylus brachymystacis*	14,767	30.6	35.2	15.1	19.1	NC_031337
Gyrodactylidea	Gyrodactylidae	*Gyrodactylus derjavinoides*	14,741	33.1	35.1	14.2	17.6	NC_010976
Gyrodactylidea	Gyrodactylidae	*Gyrodactylus gurleyi*	14,771	29.2	42.9	11.1	16.9	KU659806
Gyrodactylidea	Gyrodactylidae	*Gyrodactylus kobayashii*	14,786	29.7	41.9	11.1	17.3	NC_030050
Gyrodactylidea	Gyrodactylidae	*Gyrodactylus nyanzae*	14,885	32.2	47.9	6.7	13.2	MG970256
Gyrodactylidea	Gyrodactylidae	*Gyrodactylus parvae*	14,702	32.2	41.3	10.7	15.8	NC_031438
Gyrodactylidea	Gyrodactylidae	*Gyrodactylus salaris*	14,790	29.8	32.7	17.1	20.4	EF527269
Dactylogyridea	Diplectanidae	*Lamellodiscus spari*	14,614	31.5	45.3	7.9	15.4	MH328204
Gyrodactylidea	Gyrodactylidae	*Paragyrodactylus variegatus*	14,517	30.4	45.8	9.5	14.2	NC_024754
Tetraonchidea	Tetraonchoididae	*Paratetraonchoides inermis*	14,654	36.1	46.5	6.0	11.4	KY856918
Dactylogyridea	Diplectanidae	*Pseudorhabdosynochus yangjiangensis*	12,458	29.9	44.8	8.0	17.4	JQ038231
Dactylogyridea	Ancyrocephalidae	*Scutogyrus longicornis*	14,241	22.4	43.4	22.9	11.3	MT447060
Dactylogyridea	Dactylogyridae	*Tetrancistrum nebulosi*	13,392	27.2	38.2	13.1	21.5	NC_018031
Tetraonchidea	Tetraonchidae	*Tetraonchus monenteron*	14,791	26.2	47.2	11.2	15.4	NC_046757
Dactylogyridea	Ancylodiscoididae	*Thaparocleidus asoti*	17,493	32.1	46.6	7.3	14.1	NC_053548
Dactylogyridea	Ancylodiscoididae	*Thaparocleidus varicus*	14,088	30.1	46.8	7.6	15.5	NC_053547

**Table 2:** Nucleotide composition and skewness comparison of different elements of the mitochondrial genomes of *Capsala katsuwoni* and *Capsala martinieri*.

Region	Size (bp)	A (%)	T (%)	G (%)	C (%)	A + T (%)	AT-Skew	GC-Skew
Mitogenome	13,265/13,984	25.4/25.8	41.1/38.2	18.9/19.1	14.6/17.0	66.5/63.9	−0.24/−0.19	0.13/0.06
cox1	1563/1563	23.8/23.0	39.9/36.5	20.0/21.7	16.3/18.8	63.7/59.6	−0.25/−0.23	0.10/0.07
cox2	582/630	26.5/27.3	37.8/34.0	20.6/22.2	15.1/16.5	64.3/61.3	−0.18/−0.11	0.15/0.15
atp6	546/510	23.6/21.8	43.0/39.0	17.6/20.8	15.8/18.4	66.7/60.8	−0.29/−0.28	0.05/0.06
cox3	651/651	24.7/23.7	43.0/40.3	18.7/19.4	13.5/16.7	67.7/63.9	−0.27/−0.26	0.16/0.07
nad3	354/354	24.0/26.8	45.2/41.2	18.6/17.8	12.2/14.1	69.2/68.1	−0.31/−0.21	0.21/0.12
nad1	900/900	22.9/25.2	40.8/38.4	21.9/19.0	14.4/17.3	63.7/63.7	−0.28/−0.21	0.20/0.05
nad5	1542/1542	24.1/23.8	44.8/38.4	16.5/18.6	14.7/17.3	68.8/64.2	−0.30/−0.26	0.06/0.04
nad4	1221/1167	24.6/24.1	43.5/39.4	17.0/18.3	15.0/18.2	68.1/63.5	−0.28/−0.24	0.06/0.01
nad4L	249/249	22.5/23.7	47.4/42.6	16.9/16.9	13.3/16.9	69.9/66.3	−0.36/−0.28	0.12/0.00
nad6	453/453	21.9/22.5	45.9/43.9	19.2/19.9	13.0/13.7	67.8/66.5	−0.36/−0.32	0.19/0.18
cob	1104/1083	24.5/25.9	39.1/37.0	20.6/19.4	15.9/17.7	63.6/62.9	−0.23/−0.18	0.13/0.04
nad2	861/864	24.6/22.3	46.1/42.5	17.5/18.9	11.7/16.3	70.7/64.8	−0.30/−0.31	0.20/0.07
tRNAs	1429/1436	29.4/28.8	35.4/34.7	20.2/21.0	15.0/15.5	64.8/63.5	−0.09/−0.09	0.15/0.15
rRNAs	1662/1655	30.0/29.8	36.9/36.4	18.6/18.0	14.4/15.8	66.9/66.2	−0.10/−0.10	0.13/0.06
PCGs	10,026/9966	24.1/24.1	42.5/39.1	18.8/19.6	14.6/17.3	66.6/63.2	−0.28/−0.24	0.12/0.06

**Table 3:** Comparison of the annotated mitochondrial genomes of *Capsala katsuwoni* and *Capsala martinieri*.

Gene	Position		Size	Initiation	Termination	Anticodon	Overlapping	Intergenic	Strand
cox1	1/1	1563/1563	1563/1563	ATG/ATG	TAA/TAA			7/6	+/+
trnT	1571/1568	1635/1633	65/66			tgt/tgt			+/+
rrnL	1636/1634	2588/2576	953/943				6/10		+/+
trnC	2583/2567	2648/2634	66/68			gca/gca		1/-	+/+
rrnS	2650/2635	3364/3348	715/714				-/35	15/-	+/+
cox2	3380/3314	3961/3943	582/630	ATG/ATG	TAG/TAG			2/8	+/+
trnE	3964/3952	4030/4016	67/65			ttc/ttc			+/+
nad6	4031/4017	4483/4469	453/453	ATG/ATG	TAG/TAA			4/10	+/+
trnY	4488/4480	4548/4543	61/64			gta/gta		6/5	+/+
trnL1	4555/4549	4621/4615	67/67			tag/tag			+/+
trnS2	4622/4616	4686/4679	65/64			tga/tga		1/-	+/+
trnL2	4688/4680	4753/4745	66/66			taa/taa	-/1		+/+
trnR	4754/4745	4816/4810	63/66			tcg/tcg		2/1	+/+
nad5	4819/4812	6360/6353	1542/1542	ATG/ATG	TAA/TAG			80/80	+/+
trnG	6441/6434	6506/6499	66/66			tcc/tcc		2/2	+/+
cox3	6509/6502	7159/7152	651/651	ATG/ATG	TAA/TAA			6/1	+/+
trnH	7166/7154	7228/7216	63/63			gtg/gtg	20/-	-/2	+/+
cob	7209/7219	8312/8301	1104/1083	ATG/ATG	TAG/TAA		1/1		+/+
nad4L	8312/8301	8560/8549	249/249	GTG/ATG	TAG/TAG		28/-	-/29	+/+
nad4	8533/8579	9753/9745	1221/1167	GTG/ATA	TAA/TAG			14/15	+/+
trnF	9768/9761	9835/9823	68/63			gaa/gaa	5/3		+/+
trnM	9831/9821	9897/9887	67/67			cat/cat	36/-		+/+
atp6	9862/9888	10,407/10,397	546/510	ATG/ATG	TAG/TAG			2/6	+/+
nad2	10,410/10,404	11,270/11,267	861/864	GTG/ATG	TAA/TAG			6/18	+/+
trnD	11,551/11,286	11,616/11,351	66/66			gtc/gtc			+/+
nad1	11,617/11,352	12,516/12,251	900/900	ATG/ATG	TAA/TGA			4/29	+/+
trnV	11,277/12,281	11,342/12,344	66/64			tac/tac		11/413	+/+
trnN	12,521/12,758	12,586/12,823	66/66			gtt/gtt		6/335	+/+
trnA	11,354/13,159	11,418/13,225	65/67			tgc/tgc		60/8	+/+
trnQ	11,479/13,234	11,541/13,297	63/64			ttg/ttg		9/11	+/+
trnP	12,593/13,309	12,658/13,375	66/67			tgg/tgg	1/1		+/+
trnI	12,658/13,375	12,723/13,440	66/66			gat/gat		1/-	+/+
trnK	12,725/13,441	12,790/13,507	66/67			ctt/ctt			+/+
nad3	12,791/13,508	13,144/13,861	354/354	ATG/GTG	TAG/TAG		-/1		+/+
trnS1	13,145/13,861	13,201/13,918	57/42			gct/agc			+/+
trnW	13,202/13,919	13,265/13,984	64/66			tca/tca			+/+

## Data Availability

The genome sequence data that support the findings of this study are openly available in GenBank of NCBI at (https://www.ncbi.nlm.nih.gov, accessed on 13 April 2022/20 April 2022) under the accession numbers OL884727 and OL790148.

## Supplementary Materials

The following supporting information can be downloaded at: https://www.mdpi.com/article/10.3390/genes13081376/s1, Table S1: A+T content based on 13 mitogenomic elements of the 26 selected monogeneans; Table S2: AT skewness based on 13 mitogenomic elements of the 26 selected monogeneans; Table S3: Amino acid composition and relative synonymous codon usage of Capsala katsuwoni; Table S4: Amino acid composition and relative synonymous codon usage of Capsala martinierei.

## References

[1] Rohde K. (2005). Marine Parasitology.

[2] Bauer O.N. (1991). Spread of parasites and diseases of aquatic organisms by acclimatization: A short review. J. Fish Biol..

[3] Hutson K.S., Ernst I., Whittington I.D. (2007). Risk assessment for metazoan parasites of yellowtail kingfish *Seriola lalandi* (Perciformes: Carangidae) in South Australian sea-cage aquaculture. Aquaculture.

[4] Whittington I.D. (2004). The Capsalidae (Monogenea: Monopisthocotylea): A review of diversity, classification and phylogeny with a note about species complexes. Folia Parasit..

[5] Whittington I.D., Deveney M.R., Morgan J.A.T., Chisholm L.A., Adlard R.D. (2004). A preliminary phylogenetic analysis of the Capsalidae (Platyhelminthes: Monogenea: Monopisthocotylea) inferred from large subunit rDNA sequences. Parasitology.

[6] Chisholm L.A., Whittington I.D. (2007). Review of the Capsalinae (Monogenea: Capsalidae). Zootaxa.

[7] Boeger W.A., Kritsky D.C. (1993). Phylogeny and a revised classification of the Monogenoidea Bychowsky, 1937 (Platyhelminthes). Syst. Parasitol..

[8] Mollaret I., Jamieson B.G., Justine J.L. (2000). Phylogeny of the Monopisthocotylea and Polyopisthocotylea (Platyhelminthes) inferred from 28S rDNA sequences. Inter. J. Parasitol..

[9] Olson P.D., Littlewood D.T.J. (2002). Phylogenetics of the Monogenea–evidence from a medley of molecules. Inter. J. Parasitol..

[10] Zhang D., Li W.X., Zou H., Wu S.G., Li M., Jakovlić I., Zhang J., Chen R., Wang G.T. (2018). Mitochondrial genomes of two diplectanids (Platyhelminthes: Monogenea) expose paraphyly of the order Dactylogyridea and extensive tRNA gene rearrangements. Parasite. Vector..

[11] Kearn G.C. (1963). The egg, oncomiracidium and larval development of *Entobdella soleae*, a monogenean skin parasite of the common sole. Parasitology.

[12] Wang L., Zhou Z.H., Yuan K., Ding X.J. (2019). Three new recorded species in Capsalidae (monogenea) from fishes in the South China Sea. Acta Parasitol. Medica Entomol. Sin..

[13] Cameron S. (2014). How to sequence and annotate insect mitochondrial genomes for systematic and comparative genomics research. Syst. Entomol..

[14] Park J.K., Kim K.H., Kang S., Kim W., Eom K.S., Littlewood D.T.J. (2007). A common origin of complex life cycles in parasitic flatworms: Evidence from the complete mitochondrial genome of *Microcotyle sebastis* (Monogenea: Platyhelminthes). BMC Evol. Biol..

[15] Shao R., Barker S.C. (2007). Mitochondrial genomes of parasitic arthropods: Implications for studies of population genetics and evolution. Parasitology.

[16] Huyse T., Buchmann K., Littlewood D.T.J. (2008). The mitochondrial genome of *Gyrodactylus derjavinoides* (Platyhelminthes: Monogenea)—a mitogenomic approach for *Gyrodactylus* species and strain identification. Gene.

[17] Zhang P., Liang D., Mao R.L., Hillis D.M., Wake D.B., Cannatella D.C. (2013). Efficient sequencing of anuran mtDNAs and a mitogenomic exploration of the phylogeny and evolution of frogs. Mol. Biol. Evol..

[18] Yang C.P., Shan B.B., Liu Y., Zhao Y., Sun D.R. (2020). Next-generation sequencing yields the complete mitochondrial genome of the *Capsala pricei* Hidalgo, 1959 (Platyhelminthes: Monogenea) from South China Sea. Mitochondrial DNA Part B.

[19] Baeza J.A., Sepúlveda F.A., González M.T. (2019). The complete mitochondrial genome and description of a new cryptic species of *Benedenia* Diesing, 1858 (Monogenea: Capsalidae), a major pathogen infecting the yellowtail kingfish *Seriola lalandi* Valenciennes in the South-East Pacific. Parasite Vector.

[20] Zhang J., Wu X.Y., Li Y.W., Zhao M.W., Xie M.Q., Li A.X. (2014). The complete mitochondrial genome of *Neobenedenia melleni* (Platyhelminthes: Monogenea): Mitochondrial gene content, arrangement and composition compared with two *Benedenia* species. Mol. Biol. Rep..

[21] Yang C.P., Liu Y., Shan B.B., Zhang G.J., Zhao Y., Sun D.R., Yu W. (2021). The complete mitochondrial genome of the *Capsaloides cristatus* (Platyhelminthes, Monogenea), a pathogen of the sailfish (*Istiophorus platypterus*). Mitochondrial DNA Part B.

[22] Lohse M., Drechsel O., Bock R. (2007). OrganellarGenomeDRAW (OGDRAW): A tool for the easy generation of high-quality custom graphical maps of plastid and mitochondrial genomes. Curr. Genet..

[23] Perna N.T., Kocher T.D. (1995). Patterns of nucleotide composition at fourfold degenerate sites of animal mitochondrial genomes. J. Mol. Evol..

[24] Katoh K., Kuma K.I., Toh H., Miyata T. (2005). MAFFT version 5: Improvement in accuracy of multiple sequence alignment. Nucleic Acids Res..

[25] Capella-Gutiérrez S., Silla-Martínez J.M., Gabaldón T. (2009). trimAl: A tool for automated alignment trimming in large-scale phylogenetic analyses. Bioinformatics.

[26] Drummond A.J., Suchard M.A., Xie D., Rambaut A. (2012). Bayesian phylogenetics with BEAUti and the BEAST 1.7. Mol. Biol Evol..

[27] Darriba D., Taboada G.L., Doallo R., Posada D. (2012). jModelTest 2: More models, new heuristics and parallel computing. Nat. Methods.

[28] Kumar S., Nei M., Dudley J., Tamura K. (2008). MEGA: A biologist-centric software for evolutionary analysis of DNA and protein sequences. Brief. Bioinform..

[29] Ronquist F., Teslenko M., Van Der Mark P., Ayres D.L., Darling A., Höhna S., Larget B., Liu L., Suchard M.A., Huelsenbeck J.P. (2012). MrBayes 3.2: Efficient Bayesian phylogenetic inference and model choice across a large model space. Syst. Biol..

[30] Kang S., Kim J., Lee J., Kim S., Min G.S., Park J.K. (2012). The complete mitochondrial genome of an ectoparasitic monopisthocotylean fluke *Benedenia hoshinai* (Monogenea: Platyhelminthes). Mitochondrial DNA.

[31] Zhang D., Zou H., Jakovlić I., Wu S.G., Li M., Zhang J., Chen R., Li W.X., Wang G.T. (2019). Mitochondrial genomes of two thaparocleidus species (Platyhelminthes: Monogenea) reveal the first rRNA gene rearrangement among the neodermata. Inter. J. Mol. Sci..

[32] Zhang D., Li W.X., Zou H., Wu S.G., Li M., Jakovlić I., Zhang J., Chen R., Wang G.T. (2020). Mitochondrial genomes and 28S rDNA contradict the proposed obsoletion of the order Tetraonchidea (Platyhelminthes: Monogenea). Inter. J. Biol. Macromol..

[33] Zhang D., Zou H., Wu S.G., Li M., Jakovlić I., Zhang J., Chen R., Wang G.T., Li W.X. (2017). Sequencing of the complete mitochondrial genome of a fish-parasitic flatworm *Paratetraonchoides inermis* (Platyhelminthes: Monogenea): tRNA gene arrangement reshuffling and implications for phylogeny. Parasite Vector.

[34] Park S., Ruhlman T.A., Weng M.L., Hajrah N.H., Sabir J.S., Jansen R.K. (2017). Contrasting patterns of nucleotide substitution rates provide insight into dynamic evolution of plastid and mitochondrial genomes of Geranium. Genome Boil. Evol..

[35] Moritz C., Brown W.M. (1987). Tandem duplications in animal mitochondrial DNAs: Variation in incidence and gene content among lizards. Proc. Natl. Acad. Sci. USA.

[36] Lavrov D.V., Boore J.L., Brown W.M. (2002). Complete mtDNA sequences of two millipedes suggest a new model for mitochondrial gene rearrangements: Duplication and nonrandom loss. Mol. Biol. Evol..

[37] Rokas A., Ladoukakis E., Zouros E. (2003). Animal mitochondrial DNA recombination revisited. Trends Ecol. Evol..

[38] Bychowsky B.E. (1957). Monogenetic Trematodes, Their Classification and Phylogeny.

[39] Zhang D., Zou H., Wu S.G., Li M., Jakovlić I., Zhang J., Chen R., Wang G.T., Li W.X. (2018). Sequencing, characterization and phylogenomics of the complete mitochondrial genome of *Dactylogyrus lamellatus* (Monogenea: Dactylogyridae). J. Helminthol..

[40] Justine J.L., Lambert A., Mattei X. (1985). Spermatozoon ultrastructure and phylogenetic relationships in the monogeneans (Platyhelminthes). Inter. J. Parasitol..

[41] Šimková A., Plaisance L., Matějusová I., Morand S., Verneau O. (2003). Phylogenetic relationships of the Dactylogyridae Bychowsky, 1933 (Monogenea: Dactylogyridea): The need for the systematic revision of the Ancyrocephalinae Bychowsky, 1937. Syst. Parasitol..

