# Supplementary material for: Complete Mitochondrial Genome of Two Ectoparasitic Capsalids (Platyhelminthes: Monogenea: Monopisthocotylea): Gene Content, Composition, and Rearrangement
Source: Genes (Basel). 2022 Aug 1;13(8):1376. doi: 10.3390/genes13081376 (PMC9407395; doi:10.3390/genes13081376)
Supplement: Supplementary file 1 [file genes-13-01376-s001.zip › genes-1849395-supplementary.pdf]

Table S1. A+T content based on 13 mitogenomic elements of the 26 selected monogeneans.

Species	atp6	cox1	cox2	cox3	cytb	nad1	nad2	nad3	nad4	nad4L	nad5	nad6	rRNAs (+)	tRNAs(+)
B. seriolae	74.7	70.3	69.2	76.4	70.9	73.8	79.8	78.4	80.5	78.5	77.1	80.6	75.3	75.4
B. hoshinai	71.4	70.0	68.7	75.3	70.1	70.5	79.1	79.1	78.2	69.8	75.4	78.2	73.3	74.8
C. katsuwoni	66.7	63.7	64.3	67.7	63.6	63.7	70.7	69.2	68.1	69.9	68.8	67.8	66.9	64.8
C. martinierei	60.8	59.6	61.3	63.9	62.9	63.7	64.8	68.1	63.5	66.3	64.2	66.5	66.2	63.5
C. pricei	68.5	65.8	66.5	67.3	66.1	67.7	71.5	73.2	69.9	72.3	70.6	72.0	66.2	67.5
C. sclerosus	61.8	63.5	62.5	64.0	63.1	67.6	63.7	65.5	63.2	65.1	64.7	65.2	62.6	63.8
C. halli	61.5	62.3	60.4	62.3	62.3	63.6	63.8	65.8	62.9	66.7	62.4	N/A	65.3	63.5
D. lamellatus	67.6	67.0	63.4	66.4	68.0	68.3	70.1	71.0	69.8	71.9	71.5	74.5	68.3	68.2
E. malmbergi	67.3	66.4	65.0	66.0	66.6	69.0	69.1	69.9	67.6	69.4	69.2	72.5	67.9	71.3
G. brachymystacis	64.7	62.7	66.2	66.5	62.5	67.6	67.1	66.3	63.4	67.9	63.1	69.6	69.1	68.6
G. gurleyi	73.1	69.6	69.9	72.5	67.7	71.4	77.6	72.8	73.3	75.5	71.1	76.0	73.3	72.5
G. nyanzae	78.9	73.6	76.6	79.1	75.7	78.7	86.5	82.4	82.2	81.4	81.6	83.5	78.5	77.7
G. salaris	60.6	58.3	62.7	61.3	59.7	59.7	62.5	60.4	60.3	67.9	58.8	67.0	67.0	68.1
G. derjavinoides	70.2	65.1	66.2	68.0	63.9	68.6	71.0	70.4	67.5	74.3	66.8	73.5	70.0	69.9
G. kobayashii	73.7	68.3	69.9	72.0	67.0	72.2	75.1	73.0	72.7	77.5	71.8	76.8	73.0	72.5
G. parvae	71.6	69.8	72.2	74.6	70.0	72.8	80.0	75.2	75.6	77.1	74.6	77.4	74.5	73.2
L. spari	75.5	70.9	69.6	75.7	71.1	74.0	79.3	78.9	75.4	73.7	76.1	78.8	73.8	74.6
N. melleni	72.3	71.4	72.2	77.4	71.0	75.2	79.3	82.2	79.0	81.1	80.0	80.4	75.1	73.7
P. variegatus	78.8	71.0	72.0	75.9	71.9	74.0	82.9	78.9	76.6	77.6	77.1	80.5	75.1	73.4
P. inermis	81.2	75.1	76.9	83.1	77.6	80.9	87.9	88.1	83.8	85.4	85.8	87	81.3	82.6
P. yangjiangensis	75.5	69.8	70.7	75.0	70.9	74.1	77.0	77.7	74.4	78.9	76.2	76.7	76.9	76.2
S. longicornis	65.8	63.4	64.8	65.5	65.9	63.8	68.9	68.7	65.2	69.0	66.1	70.2	62.7	63.2
T. nebulosi	64.7	63.8	63.8	65.9	64.6	66.7	65.5	67.0	63.3	68.3	65.9	66.2	66.6	65.5
T. varicus	76.0	70.6	70.9	76.1	74.0	76.9	81.1	79.1	79.6	82.2	77.5	79.6	76.5	76.5

T. asoti	77.8	70.1	70.6	74.8	72.9	75.8	79.8	80.5	77.3	79.8	77.1	77.9	75.6	76.8
T. monenteron	72.1	69.7	68.8	72.8	71.7	72.8	76.2	77.4	72.9	73.9	73.9	75.5	71.0	71.8

Table S2. AT skewness based on 13 mitogenomic elements of the 26 selected monogeneans.

Species	atp6	cox1	cox2	cox3	cytb	nad1	nad2	nad3	nad4	nad4L	nad5	nad6	rRNAs (+)	tRNAs (+)
B. seriolae	−0.323	−0.277	−0.176	−0.289	−0.246	−0.314	−0.319	−0.309	−0.289	−0.207	−0.27	−0.295	−0.12	−0.106
B. hoshinai	−0.275	−0.281	−0.145	−0.307	−0.279	−0.26	−0.263	−0.279	−0.28	−0.236	−0.254	−0.312	−0.113	−0.096
C. katsuwoni	−0.29	−0.25	−0.18	−0.27	−0.23	−0.28	−0.3	−0.31	−0.28	−0.36	−0.3	−0.36	−0.1	−0.09
C. martinierei	−0.11	−0.19	−0.23	−0.28	−0.18	−0.21	−0.31	−0.26	−0.24	−0.28	−0.26	−0.32	−0.1	−0.09
C. pricei	−0.32	−0.29	−0.18	−0.32	−0.27	−0.30	−0.29	−0.20	−0.29	−0.28	−0.33	−0.37	−0.13	−0.14
C. sclerosus	−0.371	−0.32	−0.172	−0.406	−0.29	−0.35	−0.4	−0.439	−0.399	−0.44	−0.273	−0.359	−0.114	−0.116
C. halli	−0.242	−0.273	−0.116	−0.358	−0.273	−0.303	−0.271	−0.406	−0.268	−0.425	−0.274	N/A	−0.065	−0.08
D. lamellatus	−0.322	−0.291	−0.238	−0.313	−0.288	−0.356	−0.324	−0.271	−0.366	−0.318	−0.306	−0.309	−0.049	−0.12
E. malmbergi	−0.382	−0.265	−0.16	−0.336	−0.312	−0.276	−0.37	−0.325	−0.343	−0.394	−0.217	−0.265	−0.057	−0.108

G. brachymystacis	−0.181	−0.174	−0.096	−0.111	−0.088	−0.187	−0.117	−0.167	−0.1	−0.148	−0.078	−0.089	−0.004	−0.008
G. gurleyi	−0.301	−0.313	−0.184	−0.266	−0.23	−0.315	−0.245	−0.344	−0.228	−0.277	−0.226	−0.215	−0.077	−0.076
G. nyanzae	−0.328	−0.254	−0.256	−0.277	−0.255	−0.31	−0.242	−0.26	−0.214	−0.219	−0.224	−0.281	−0.057	−0.086
G. salaris	−0.119	−0.109	−0.063	−0.097	−0.083	−0.166	−0.069	−0.16	−0.071	−0.16	−0.058	−0.086	0.01	−0.026
G. derjavinoides	−0.122	−0.094	−0.075	−0.041	−0.048	−0.09	−0.067	−0.122	−0.037	−0.135	−0.029	−0.076	0.001	−0.01
G. kobayashii	−0.296	−0.25	−0.17	−0.226	−0.216	−0.257	−0.276	−0.323	−0.205	−0.223	−0.208	−0.213	−0.054	−0.076
G. parvae	−0.221	−0.22	−0.152	−0.203	−0.157	−0.245	−0.137	−0.288	−0.149	−0.229	−0.131	−0.155	−0.055	−0.046
L. spari	−0.375	−0.286	−0.249	−0.331	−0.3	−0.295	−0.281	−0.162	−0.292	−0.298	−0.242	−0.243	−0.073	−0.083
N. melleni	−0.251	−0.246	−	−	−	−0.301	−0.205	−0.21	−0.233	−0.228	−0.2	−0.261	−0.057	−0.078

			0.135	0.263	0.251									
P. yangjiangensis	−0.279	−0.266	−0.159	−0.365	−0.259	−0.276	−0.279	−0.245	−0.264	−0.216	−0.145	−0.223	−0.027	−0.07
P. variegatus	−0.272	−0.262	−0.222	−0.295	−0.196	−0.332	−0.287	−0.3	−0.279	−0.263	−0.251	−0.255	−0.084	−0.112
P. inermis	−0.232	−0.193	−0.188	−0.213	−0.181	−0.226	−0.193	−0.165	−0.165	−0.246	−0.139	−0.177	0.035	0.013
S. longicornis	−0.465	−0.309	−0.302	−0.438	−0.363	−0.392	−0.402	−0.447	−0.408	−0.516	−0.383	−0.496	−0.104	−0.165
T. varicus	−0.297	−0.32	−0.216	−0.311	−0.292	−0.298	−0.348	−0.247	−0.322	−0.304	−0.262	−0.303	−0.019	−0.067
T. asoti	−0.29	−0.293	−0.202	−0.372	−0.297	−0.29	−0.293	−0.233	−0.314	−0.274	−0.256	−0.328	−0.066	−0.057
T. nebulosi	−0.315	−0.235	−0.138	−0.204	−0.22	−0.229	−0.218	−0.207	−0.248	−0.282	−0.165	−0.26	−0.012	−0.081
T. monenteron	−0.43	−0.325	−0.264	−0.399	−0.352	−0.391	−0.464	−0.365	−0.443	−0.461	−0.281	−0.392	−0.114	−0.084

Table S3. Amino acid composition and relative synonymous codon usage of *Capsala katsuwoni.*

Codon	Count	RSCU	Codon	Count	RSCU	Codon	Count	RSCU	Codon	Count	RSCU
UUU(F)	275	1.57	UCU(S)	75	2.01	UAU(Y)	163	1.48	UGU(C)	71	1.39
UUC(F)	75	0.43	UCC(S)	35	0.94	UAC(Y)	58	0.52	UGC(C)	31	0.61
UUA(L)	181	2.19	UCA(S)	40	1.07	UAA(*)	65	1.04	UGA(W)	49	0.84
UUG(L)	89	1.07	UCG(S)	11	0.3	UAG(*)	60	0.96	UGG(W)	68	1.16
CUU(L)	100	1.21	CCU(P)	50	2.02	CAU(H)	33	0.99	CGU(R)	12	1.55
CUC(L)	28	0.34	CCC(P)	23	0.93	CAC(H)	34	1.01	CGC(R)	8	1.03
CUA(L)	58	0.7	CCA(P)	19	0.77	CAA(Q)	29	1.29	CGA(R)	2	0.26
CUG(L)	41	0.49	CCG(P)	7	0.28	CAG(Q)	16	0.71	CGG(R)	9	1.16
AUU(I)	104	1.49	ACU(T)	60	1.78	AAU(N)	44	1.31	AGU(S)	53	1.42
AUC(I)	36	0.51	ACC(T)	18	0.53	AAC(N)	23	0.69	AGC(S)	20	0.54
AUA(M)	107	1.25	ACA(T)	47	1.39	AAA(K)	61	1.26	AGA(S)	29	0.78
AUG(M)	64	0.75	ACG(T)	10	0.3	AAG(K)	36	0.74	AGG(S)	35	0.94
GUU(V)	127	1.67	GCU(A)	51	1.98	GAU(D)	58	1.57	GGU(G)	90	2.18
GUC(V)	29	0.38	GCC(A)	19	0.74	GAC(D)	16	0.43	GGC(G)	15	0.36
GUA(V)	85	1.11	GCA(A)	19	0.74	GAA(E)	33	1.18	GGA(G)	25	0.61
GUG(V)	64	0.84	GCG(A)	14	0.54	GAG(E)	23	0.82	GGG(G)	35	0.85

Table S4. Amino acid composition and relative synonymous codon usage of *Capsala martinierei.*

Codon	Count	RSCU	Codon	Count	RSCU	Codon	Count	RSCU	Codon	Count	RSCU
UUU(F)	226	1.41	UCU(S)	93	2.11	UAU(Y)	132	1.4	UGU(C)	61	1.23
UUC(F)	94	0.59	UCC(S)	53	1.2	UAC(Y)	57	0.6	UGC(C)	38	0.77
UUA(L)	129	1.82	UCA(S)	44	1	UAA(*)	73	1.09	UGA(W)	44	0.77
UUG(L)	70	0.99	UCG(S)	16	0.36	UAG(*)	61	0.91	UGG(W)	70	1.23
CUU(L)	99	1.39	CCU(P)	56	1.91	CAU(H)	35	1.19	CGU(R)	10	1.38
CUC(L)	35	0.49	CCC(P)	22	0.75	CAC(H)	24	0.81	CGC(R)	8	1.1
CUA(L)	54	0.76	CCA(P)	27	0.92	CAA(Q)	30	1.15	CGA(R)	4	0.55
CUG(L)	39	0.55	CCG(P)	12	0.41	CAG(Q)	22	0.85	CGG(R)	7	0.97
AUU(I)	72	1.47	ACU(T)	43	1.48	AAU(N)	36	1.18	AGU(S)	54	1.22
AUC(I)	26	0.53	ACC(T)	23	0.79	AAC(N)	25	0.82	AGC(S)	25	0.57
AUA(M)	97	1.19	ACA(T)	38	1.31	AAA(K)	72	1.4	AGA(S)	33	0.75
AUG(M)	66	0.81	ACG(T)	12	0.41	AAG(K)	31	0.6	AGG(S)	35	0.79
GUU(V)	88	1.36	GCU(A)	65	1.9	GAU(D)	39	1.13	GGU(G)	55	1.35
GUC(V)	33	0.51	GCC(A)	33	0.96	GAC(D)	30	0.87	GGC(G)	30	0.74
GUA(V)	88	1.36	GCA(A)	26	0.76	GAA(E)	40	1.13	GGA(G)	26	0.64
GUG(V)	50	0.77	GCG(A)	13	0.38	GAG(E)	31	0.87	GGG(G)	52	1.28
